# The development of a physical therapy service to treat urinary incontinence: Results of a RE-AIM evaluation

**DOI:** 10.3389/fgwh.2022.1004140

**Published:** 2022-10-25

**Authors:** Elisa Brosina de Leon, Maria Natália Cardoso, Elorides de Brito, Maira Mendes dos Santos, Fabio Araujo Almeida

**Affiliations:** ^1^Programa de Pós Graduação Stricto Sensu em Ciências do Movimento Humano, Faculdade de Educação Física e Fisioterapia, Universidade Federal do Amazonas, Manaus, Brazil; ^2^Faculdade de Educação Física e Fisioterapia, Universidade Federal do Amazonas, Manaus, Brazil; ^3^Fundação Universidade Aberta da Terceira Idade, Manaus, Brazil; ^4^College of Public Health, Department of Health Promotion, University of Nebraska Medical Center, Omaha, NE, United States

**Keywords:** RE-AIM, pelvic care, urinary incontinence, physical therapy, older women, community health care, health services administration

## Abstract

**Background:**

A conservative physiotherapy service development addressed to treat urinary incontinence for older women was studied using the RE-AIM (reach, effectiveness, adoption, implementation, and maintenance) framework.

**Design:**

We conducted a pragmatic case study design based on RE-AIM.

**Settings/participants:**

Included women ≥ 60 years of age, with self-reported UI symptoms.

**Results:**

A total of 34 older women were enrolled in the service with a mean age of 61.53 years. There was a significant improvement in the strength of the pelvic floor muscles, power, endurance, and fast contraction capacity after the intervention, however, it was observed a high dropout rate. Program implementation was supported by Physical Therapy teams who engaged in care coordination. The program has been maintained for over 4 years.

**Conclusion:**

Our findings demonstrate that UI patients would benefit from physiotherapy treatment and that this intervention is feasible. This RE-AIM evaluation provides lessons learned and strategies for future adoption, implementation, and maintenance of a Physical Therapy pelvic service.

## Introduction

Urinary incontinence (UI) is defined as any involuntary leakage of urine (1). The main types of UI are stress, urgency, and mixed, with stress being most prevalent (2). Urinary incontinence is associated with substantial costs. Women spent nearly R$3.750,00 per year out of pocket for incontinence management, had a significant decrement in quality of life, and were willing to pay nearly R$7.000,00 per year for cure (3). Direct costs fall into categories of cost of management by the individual or caregivers, supplies, and treatment (ranging from education and exercise to drugs and surgery), and costs related to lost wages by affected individuals and their caregivers (4).

Despite UI not being inherent to aging, prevalence increases in women aged 70 years and older (5). UI affects emotional wellbeing more than anything else. Half to one-third of the patients experienced higher levels of depression and stress and lower levels of self-esteem (6), which may contribute to sexual dysfunction (7). Individuals of all ages are faced with physical, emotional, sexual and financial changes (4). The existing literature specifies surgery as a recurrent treatment with an overall lifetime rate of reoperation of about 8–9% (8, 9).

Conservative management should be the starting point for any patient with UI. Conservative therapies are recommended as they can be well accepted (10) effective, well-tolerated, and are usually associated with the least risk of harm (9, 11), regardless of the intervention protocol used (12–14). Among the conservative treatment, pelvic floor muscle training (PFMT) is recommended as a first-line treatment for UI: level 1 evidence (15, 16). However, UI care is still scarce, especially related to conservative treatment in Brazil.

Therefore, in 2018, through the interinstitutional partnership between the Polyclinic Coordinator of the Fundação Universidade Aberta da Terceira Idade (FUnATI), the Academic League of Physical Therapy in Women's Health (LAFISM), and the Physical Therapy Program of the Faculty of Physical Education and Physical Therapy (FEFF) of the Federal University of Amazonas (UFAM), the idealization and implementation of a Pelvic Physical Therapy service for specialized care for women with UI in Amazonas, Brazil were established, beginning its activities in April 2018. This study aims to evaluate the development process of a Pelvic Physical Therapy Service for older women, using the RE-AIM framework.

## Methods

### Design

This study is a pragmatic case study design. Evaluation research was carried out using a structured assessment to analyze all resources committed to the Pelvic Physical Therapy Project. The data was collected from August 2018 to December 2019. The research was conducted at FUnATI's Gerontological Polyclinic in Amazonas.

The study protocol was approved by Ethics Committee from Amazonas Federal University (number 3.670.863) and all methods were performed following relevant guidelines and regulations (Declaration of Helsinki). All participants provided written informed consent.

Eligibility criteria included women ≥ 60 years of age, with self-reported UI symptoms, including Stress UI, Urge UI, or Mixed. Women with neurogenic bladder, gynecological, or urinary tract infections, infectious diseases, pelvic organ prolapse grade >2, or any comorbidities or risk factors that could compromise the treatment were excluded. Women who disclaimed physical examination and 3 or more absences in the treatment were excluded from the effectiveness analysis.

### Intervention description

**Figure 1:**
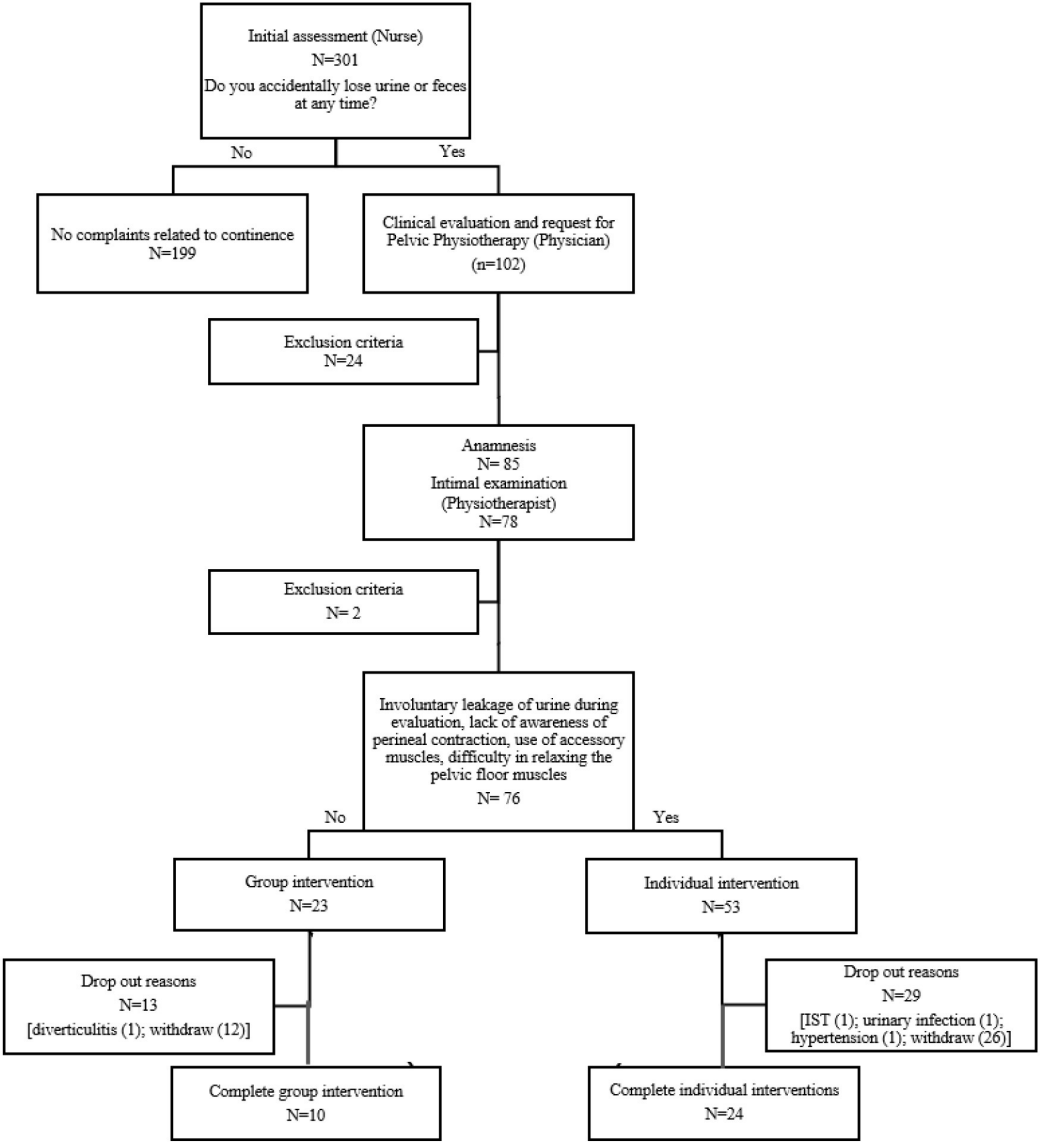
Flow chart.

The flow established by the service begins with the initial assessment. It consists of collecting personal data (reception). Anthropometric, and blood pressure measurements by a nursing technician, and clinical information by a nurse. Clinical information includes age, sphincter continence, which encompasses the question “Do you accidentally lose urine or feces at any time?” (17). After screening, patients were assisted by the physician and referred to Physical Therapy evaluation (Figure 1).

Health history tracking begins by asking the patient a simple question: “Have you leaked urine in the past 3 months?” If she responds yes, then the physical therapist asks whether the leakage is associated with impact such as jogging or coughing (stress UI) or whether it is accompanied by a strong feeling of urgency, even leaking, when the patient approaches the bathroom (urge UI) or mixed. Other variables were included in the anamnesis as previous pregnancy, the number of deliveries, cesarean section, and vaginal delivery, types of UI (Stress UI, Urge UI, Mixed), and time of symptoms (months). The physical examination performed by a certified physical therapist included abdominal, pelvic, and perineal examinations (18).

Functional assessment of pelvic floor muscle strength was performed by digital muscle test (AFA) (19). The exam followed the classification, grade 0: when there was no objective perineal function; grade 1: recognizable only on palpation; grade 2: weak contraction on palpation; grade 3: opposing resistance to palpation, not maintained; and grade 4: opposing resistance > 5 sec (20).

The evaluation of the contractile components of the pelvic floor muscles was measured by PERFECT. PERFECT is an acronym to remind all health professionals of the need to assess the main components of pelvic floor muscles contractility (21). This is a protocol evaluated PERFECT scheme, which includes assessments of the power (P), endurance (E), number of repetitions (R), and number of fast (1 second), contractions (F). Additionally, every (E) contraction (C) was timed (T). The power was graded from 0 to 5, according to the Oxford grading system (22).

The measurement of urinary loss was quantified by the Pad Test (23). The purpose of the test was to assess the volume of urinary loss through the use of a pad. Urinary losses were evaluated using the 1 h protocol and classified as follows: losses of up to 1 g were considered insignificant; between 1.1 and 9.9 g were classified as mild losses; between 10 and 49.9 g they were moderate losses; and above 50 g, severe losses (24). The pad was measured before and after the test.

Considering that individual and group-based treatment could improve UI symptoms (25) and aiming to increase service capacity to assist more patients, following Physical Therapy evaluation, patients were assigned to one of two treatment groups (individual or group-based). After the completion of sessions, all patients completed the same assessment protocol as before treatment initiation performed by the same physical therapist.

The assessment evaluation allowed stratification into two groups: individual or group-based intervention, as shown in Figure 1. The criteria adopted to indicate individual care included: (1) involuntary leakage of urine during evaluation, or (2) lack of awareness of perineal contraction, or (3) use of accessory muscles (abdominal, gluteal, and hip adductors muscles) even after orientation, or (4) difficulty in relaxing the pelvic floor muscles. Patients who did not meet these criteria were referred to group-based intervention. All patients in both groups received 10 intervention sessions with additional sessions being available (up to 10 more for a total of 20 sessions) dependent upon progress being made.

Individual treatment: Patients in the individual treatment underwent Pelvic floor muscle training (PFMT). At the first visit, patients learned how to contract the PFMs correctly without contracting the adjacent muscles, such as the abdominal, gluteal, and hip adductor muscles, with verbal instruction and palpation of the perineal body (26). During the sessions, muscle stretching and hip mobility exercises were performed, exercises to strengthen the pelvic floor muscles, all associated with diaphragmatic breathing.

Additionally, patients received Physical Therapy follow-up, twice a week, lasting 45 min, until discharge prescription. The service was open from Tuesdays to Fridays from 8 am to 12 pm. Interns offered their assistance on Wednesdays and Fridays, always under the supervision of the physical therapist. Evaluations of new patients were determined by the waiting list organized by the administrative sector of the Polyclinic, whenever there was the availability of new places.

Group-Based: Patients allocated to the group performed PFMT as previously described. Additionally, patients in this group received the same Physical Therapy follow-up as described in the individual treatment group.

All patients were reassessed every 10 sessions following the same protocol and physical examination presented above. If there was no significant clinical improvement, 5 more sessions were requested, up to a maximum of 20 sessions per patient. After discharge, patients were asked to record their perceptions about the entire Physical Therapy treatment and the service provided in a letter, allowing for a qualitative assessment of the project.

### The RE-AIM framework

To evaluate implementation, the RE-AIM model was used. The RE-AIM framework helps practitioners ask important questions during program planning, implementation, dissemination, and evaluation (27). RE-AIM is an acronym consisting of five elements or dimensions: Reach, Effectiveness, Adoption, Implementation, and Maintenance (28).

Reach: as applied to health policy, it is the absolute number, proportion, and representativeness of individuals who are willing to participate in a program, or those whose health is to be improved as a result of policy (29). We assessed Reach using data from the target population, the participation rate, and dropouts. To calculate the participation rate, the denominator was the number of patients who had declared UI symptoms, and the numerator was the number of patients attended by the service. We also included costs for recruitment until the patient is enrolled in Physical Therapy service (all the steps to reach the specific service, including management/ front desk gal/ nursing attendance) according to the average salary in Amazonas (wage level/hour) for each position and their respective working time (30).

Effectiveness addresses the impact of an intervention on important outcomes when tested under ideal conditions or in real-world environments by individuals who are not part of the research team (29). Effectiveness in this study was defined as the comparison of results measured before and after the intervention (individual or group interventions) by applying AFA, PERFECT, and PAD TEST. Cost analysis was also included in this dimension. Cost analysis considered the wage level/hour of a physical therapist in Amazonas (30) and the average number of visits received by patients until discharge (wage level/ number of visits).

At the organizational level, adoption is measured by the absolute number, representativeness of environments, and intervention agents (people who deliver the program) who are willing to start a program (29). Adoption in this study was defined by the number of employees involved in the implementation of the service and the description of the necessary professional skills.

Implementation refers to the extent to which a program is delivered as intended (29). In this study, implementation describes the frequency of contact, the average number of sessions held until discharge, the physical space, as well as the material and human resources needed to carry out the services.

Maintenance refers to long-term sustainability at both the configuration and individual levels. It also encompasses the insurance of long-term benefits and institutionalization of intervention and ongoing community capacity for implementation (29). In this study, the continuity of the program and the monthly cost of its maintenance were evaluated.

### Data analysis

Data were presented as mean ± standard deviation and percentage. The statistical evaluation of the assessment and reassessment data of the AFA, PERFECT, and Pad Test measures was performed by applying the Kolmogorov-Smirnov normality test, followed by the Student *t*-test for repeated measures, considering a significant *p* ≤ 0.05.

## Results

The final sample consisted of 34 women with an average age of 63.1 ± 0.23 years and an average BMI of 29.12 ± 0.98. Student's *t-*tests indicated that the individual and group intervention did not differ significantly on any of the following variables: age, BMI, number of pregnancies, vaginal delivery, and cesarean section. Detailed sample characteristics are listed in Table 1.

**Table 1:** Sample characteristics of participants in intervention study.

**Variable**	**Individual (*n =* 24)**	**Group-based** **(*n =* 10)**	**Total (*n =* 34)**	***p***
Age (average ± st)	62.42 ± 2.16	64.40 ± 4.32	61.53 ± 0.23	0.16
Body mass index (Kg/cm^2^)	28.8 ± 6.84	27.05 ± 4.81	29.12 ± 0.98	
Pregnancy	3.42 ± 2.80	4.3 ± 3.77	3.68 ± 3.08	0.45
Delivery	2.71 ± 2.11	3.60 ± 3.24	3.04 ± 2.56	0.19
Cesarean Section	0.55 ± 0.74	0.9 ± 1.10	0.66 ± 0.87	0.29
Vaginal Delivery	2.35 ± 2.39	2.70 ± 3.68	2.45 ± 2.78	0.74
Types of UI (*n*; %)				
Stress UI	12; 50%	6; 60%	18 (%)	
Urge UI	2; 8.33%	1; 10%	3 (10%)	
Mixed UI	10; 41.77%	3; 30%	13(32%)	
Number of assistance	405	189	594	
Average of assistence/pacient	16.87	18.90	17.47	
Time of symptoms (months)	30.0 ± 32.33	40.67 ± 25.68	45.36 ± 10.60	0.39

st, standard deviation. t, Student test.

### Reach

A total of 102 women declared they had UI symptoms with 23.5% (*n* = 24) being excluded (Figure 1). All women who were referred to the service by a physician were invited to participate in the study (*n* = 85) with 7 declining the pelvic examination and 2 not meeting eligibility criteria. Therefore, the participation rate was 74.5%.

The recruitment cost calculated in this research referred to the professionals needed to carry out the initial assessment of patients and referral based on the clinical complaint presented. The total amount of human resources was R$ 3.037.24, considering front desk clerk (R$ 5.76/h; R$ 1.064.50/40hs), nursing technicians (R$ 6.69/h; R$ 842.94/30hs), and nurse (R$ 13.45/h; R$ 1.129.80/20hs). It is important to mention that said cost was not specific to pelvic service. However, they were essential within the screening flow created.

As shown in Figure 1, the original sample was 78 women; 43,6% were lost to follow-up. Dropout was similar in both groups (individual intervention = 54,71%; group intervention = 56,5%). Of those who dropped out, two participants had acute chronic disease, 2 had non-sexually transmissible diseases and urinary infections. Five dropped out after performing the pelvic evaluation and 38 patients were excluded after 3 unexcused absences.

### Effectiveness

As shown in Table 2, the effects of time in both groups group were significant in all measurements. There was a significant improvement (*p* < 0.05) in the strength of the pelvic floor muscles, power, endurance, and fast contraction capacity comparing data from before and after the intervention. Also, there was a significant decrease in the amount of urinary loss. There were no significant differences between intervention groups (individual vs. group-based).

**Table 2:** Results of repeated-measures ANOVAs measuring (*n* = 34).

**Variables**	**Before intervention**		**After intervention**		**Time**	**p**	**Intervention**	**p**
	**Individual**	**Group**	**Individual**	**Group**				
AFA	2.42 ± 0.58	2.60 ± 0.97	3.14 ± 0.99	3.38 ± 0.92	F_(1, 29)_ = 17.54	0.0002	F _(1, 28)_ = 1.016	0.3222
PERFECT								
Power	2.43 ± 0.59	2.80 ± 1.14	3.55 ± 0.86	3.00 ± 0.58	F _(1, 27)_ = 22.59	< 0.0001	F_(1, 26)_ = 0.06818	0.7961
Endurance	3.50 ± 1.84	3.79 ± 2.60	6.64 ± 3.36	6.29 ± 3.04	F_(1, 28)_ = 14.01	0.0008	F_(1, 27)_ = 0.01279	0.9108
Repetitions	4.30 ± 1.57	4.54 ± 2.81	7.23 ± 3.19	6.00 ± 3.74	F_(1, 28)_ = 8.396	0.0072	F_(1, 27)_ = 0.5112	0.4807
Fast contractions	3.20 ± 2.15	3.88 ± 3.40	7.80 ± 4.54	7.00 ± 4.47	F_(1, 28)_ = 25.69	< 0.0001	F_(1, 27)_ = 0.3425	0.5632
Urinary loss	6.57 ± 11.46	5.14 ± 6.94	2.73 ± 5.04	0.5 ± 0.97	F_(1, 24)_ = 9.580	0.0049	F_(1, 23)_ = 0.04628	0.8316

*p* < 0.05; AFA, pelvic floor muscle strength; PERFECT, contractile components of the pelvic floor muscles.

The cost for maintaining the professional activities proposed concerning clinical improvement until discharge was R$ 389.59/patient. However, this is not the actual cost passed on to the FUnATI service since the partnership established with UFAM allowed the expansion of care capacity.

### Adoption

At the organizational level, the Pelvic Physical Therapy Service was implemented only at the FUnATI Polyclinic. A cooperation agreement had already been established between the institution and the University, enabling the establishment of care with a reduction in operating costs, so institutional participation was 100%. Two professionals were linked to the service, one as an experienced pelvic floor physiotherapist and a professor at the UFAM Physical Therapy College. As for the participation of Physical Therapy students, 5 positions were offered to volunteers and 1 position for a scholarship holder (extension scholarship). It's worthy of note that the physical therapist responsible for the service must have specific skills and be properly trained to conduct assistance.

### Implementation

All patients received Physical Therapy follow-up, individually or in groups, twice a week, lasting 45 min, until discharge prescription. The service was open from Tuesdays to Fridays from 8 am to 12 pm. The interns helped out on Wednesdays and Fridays, always under the supervision of the physical therapist. Physiotherapeutic evaluations of new patients were determined by the waiting list organized by the administrative sector of the service, whenever new places were available. The average number of sessions held until discharge was 18.9 sessions for group intervention and 16.87 sessions for individual ones. The pelvic Physical Therapy room was 15 m^2^. A reception, screening room, a preliminary nursing evaluation room, and a physician's evaluation room also integrated the service flow.

UFAM was the main sponsor for the Physical Therapy room materials. The purchase of consumables, detailed in Table 3, was shared between FUnATI and the funds received from the project's approval in UFAM's Extension Curriculum Activity Program, summing up to R$ 1,500.00. Other permanent materials were loaned with caution by UFAM. FUnATI provided the necessary physical space for the activities and UFAM allocated the teacher's workload and students to act in this project.

**Table 3:** Description of the materials used for the implementation of the pelvic physical therapy service.

**Product**	**Quantitaty**	**Unity value**	**Total**
**Permanent products**			
Digital scale	1	R$ 864.15	R$ 864.15
Precision scales	1	R$ 19.90	R$ 19.90
Professional gym ball (65 cm)	6	R$ 119.98	R$ 359.94
Mat	10	R$ 19.90	R$ 199.00
Curtain	3	R$ 71.73	R$ 215.19
Mirror 200 × 150 cm	1	R$ 259.90	R$ 259.90
Tape measure	1	R$ 3.89	R$ 3.89
Gurney	3	R$ 369.9	R$ 1.109.7
Dumbbell 1 kg	3	R$ 5.40	R$ 16.20
Dumbbell 2 kg	3	R$ 13.00	R$ 39.00
Staircase with wooden handrail	1	R$ 1.482.00	R$ 1.482.00
Wall bars	1	R$ 548.91	R$ 548.91
Total			**R$ 5,117.78**
**Consumption products**			
Resistance bands	6	R$ 8.31	R$ 49.90
Gloves	1 (1000 un)	R$ 164.90	R$ 164.90
Masks	1 (100 un)	R$ 6.00	R$ 6.00
Cap	1 (100 un)	R$ 13.49	R$ 13.49
Aquaglide water-based Lubrication gel	1 (250mg)	R$ 7.70	R$ 7.70
Pad	2 (32 un)	R$ 11.00	R$ 22.00
Total			**R$ 256.98**
**TOTAL**			**R$ 5,370.76**

Workload organization was distributed as follows: physical therapist, 20 h/week; 1 extension scholarship, 8 h/week; student volunteers, 8 h/week; professor, 2 h/week. As for remuneration, the total invested for payment of human resources provided was R$ 1.503.00 (Physical therapist R$ 22.36/h; R$ 1.503.00 /16 h). The extension fellow receives the amount of R$ 400.00/month, (*n* = 5), while student volunteers and the teacher do not receive remuneration for the service.

### Maintenance

The project was the driving force behind the implementation of the Pelvic Physical Therapy Service with free care for SUS users. This service is closely related to the institutional objective: “to contribute to improving the levels of physical-mental and social health of the older adults, using the possibilities existing in the university institution, through research, gerontological education and social wellbeing” and has been kept active by the institution.

The costs for maintaining the service are based on the sum of human resources (physical therapist = R$ 1.503.00) and the monthly purchase of consumables (R$ 252.98) totaling R$ 1.755.98.

## Discussion

To our knowledge, this is the first study that described the process of Pelvic Physical Therapy Service implementation based on the RE-AIM model. The RE-AIM analysis demonstrates how additional support and specific care practices can benefit women who suffer from UI, increasing their knowledge, decreasing symptoms, and improving self-satisfaction. Reach, Effectiveness, Adoption, Implementation, and Maintenance dimensions were fully described allowing a better understanding of the main needs of this specialized service. The findings of this study clearly show the feasibility of a specialized implementation to assist UI patients.

In general, there is a lack of pelvic Physical Therapy services tailored to fit the unique needs of patients with UI who experience distressing symptoms. Considering demographic projections, human resources, and health system constraints, there is a pressing need to scale up evidence of low-cost interventions in urinary incontinent aging women and thereby address the clinical, functional, and social aspects of this subpopulation's highly prevalent condition (25). The results of this study will be relevant to clinicians and decision-makers.

Specialized pelvic programs with protocols focused on conservative treatment are likely to be effective for older women (15). The results before and after intervention were significantly high in both groups in our study. Although no standardized protocols were applied, kinesiotherapy and pelvic exercises proved to be very effective in reducing symptoms related to UI, achieving similar results to other randomized clinical studies (9, 26, 31).

In addition to this, individual treatment did not show superiority compared to group intervention in aging women with UI. Group-based physiotherapy seems to be equally effective, as well as more cost-effective, compared to individual one-on-one sessions, impacting positively on the accessibility of continence care (25). However, Janssen et al. (32) reported many women had no improvement after group treatment, emphasizing that an important factor to predict the success of therapy is to better select patients who might benefit most (32). This initiative highlights the need for an individualized recommendation of the type of treatment to be used, individual or group, based on a specialized evaluation. This was the approach we used for this study.

Even with individualized recommendations, there is a risk of patient dropout in Physical therapy. In studies by Dumoulin et al. (25) and Kim et al. (31), both randomized clinical trials, the dropout rate was not a problem, being 6.9 and 2.36%, respectively (25, 31). The high dropout rate observed in our study differed from theirs. An important difference observed in Dumoulin's study was the orientation of performing the exercises at home in addition to the treatment performed in the clinic. Adherence to the home exercise program was assessed through participants' exercise diaries and by telephone (25). Probably, the higher frequency of performing exercises resulted in greater self-efficacy.

One of the reasons for droping out is most of the women who tend to decline to be involved in UI treatment did not feel they need therapy because they have mild symptoms of urinary incontinence or are not bothered by their wet episodes. Another frequently mentioned reason is that women have other higher priorities such as their jobs/activities and/or taking care of important others (33). Also, the dropout rate can be influenced by low literacy in pelvic physiotherapy. Despite the recognition of the complaint, some users neglect UI as a disease (34), and do not adhere to the treatment. Furthermore, we may still deal with the prejudice related to UI.

Older people who suffer from UI tend to realize that this is a condition caused by old age and that advanced age is a barrier to effective treatment. An alternative explanation for treatment dropout may be related to the natural history of UI and patterns of self-management. Incontinence usually has a gradual onset, and many women have experienced the condition for many years before they seek treatment (35). Furthermore, in our study, fear and stigma of the pelvic exam might have interfered with adherence. In future studies, the inclusion of health education strategies (36) may generate greater interest and motivation for participation in the treatment.

Our study has some limitations. First, since UI symptoms were self-reported, it is likely that we may have over- or underestimated the prevalence. However, the validity of self-reported UI, compared with clinical diagnosis, has been established in previous research (6). Second, this study was carried out at the community level, with the prediction that it would be implemented as a permanent program. Even though we did not apply a straight treatment protocol during the data collection, patients reported significantly improved symptoms.

As the strongest strength we emphasize the interinstitutional partnership between FUnATI and UFAM which allowed the expansion of the care capacity of the Pelvic Physiotherapy Service The partnership was a great strategic possibility because it allowed the rational use of the financial and human resources necessary for the implementation. In addition, the intern's participation increased service capacity and also lead to a valuable learning environment. The partnership between the public sector and the service (university and service) has also shown to be very advantageous for both patients and students, allowing for a learning environment and increasing the capacity of assistance.

## Conclusion

The physiotherapy intervention showed a significant improvement in the strength of the pelvic floor muscles, power, endurance and fast contraction capacity, however, it was observed a high dropout rate. After the conclusion of the study the service was maintained by the institution demonstrating it is feasible. This evaluation of a Physical Therapy Pelvic Service using the RE-AIM framework provides insights for dissemination to other gerontology programs and provides a model for specialized care for patients in other age groups. Our findings demonstrate that patients with UI may benefit from the effectiveness of conservative treatment.

## Data availability statement

The raw data supporting the conclusions of this article will be made available by the authors, without undue reservation.

## Ethics statement

The studies involving human participants were reviewed and approved by UFAM. The patients/participants provided their written informed consent to participate in this study.

## Author contributions

EBL is the principal investigator and has directed the planning of the study design and developed assessment forms, study interventions, and database. She is in charge of data management and will take part in the analysis and interpretation of data in addition to the submission of the reports for publication. She has the authority over the project. MC took part in the implementation and writing. EB and MdS participated in the design phase. FA participated in the analysis and interpretation of data and drafted the manuscript. All authors have done critical revision of the manuscript and approved the final version.

## Funding

This study was financed in part by the Coordenação de Aperfeiçoamento de Pessoal de Nível Superior – Brasil (CAPES) – Finance Code 001, Conselho Nacional de Pesquisa (CNPq) – PIBIC Grant and Federal University of Amazonas (UFAM) – PACE Grant.

## Conflict of interest

The authors declare that the research was conducted in the absence of any commercial or financial relationships that could be construed as a potential conflict of interest.

## Publisher's note

All claims expressed in this article are solely those of the authors and do not necessarily represent those of their affiliated organizations, or those of the publisher, the editors and the reviewers. Any product that may be evaluated in this article, or claim that may be made by its manufacturer, is not guaranteed or endorsed by the publisher.

## Abbreviations

UI: Urinary Incontinence
ICS: International Continence Society
SUS: Unified Health System
FUnATI: Fundação Universidade Aberta da Terceira Idade
LAFISM: Academic League of Physical Therapy in Women's Health
FEFF: Physical Therapy Program of the Faculty of Physical Education and Physical Therapy
UFAM: Federal University of Amazonas
IVCF-20: Index of Clinical Functional Vulnerability-20
BMI: Body mass index
AFA: Functional assessment of pelvic floor muscle
PERFECT: (P) Power, (E) Endurence, (R) Repetitions, (F) Fast contractions
ST: Standard deviation. t Student test.
